# The effects of a combination treatment with mesenchymal stem cell and platelet-rich plasma on tendon healing: an experimental study

**DOI:** 10.3906/sag-2105-145

**Published:** 2021-10-17

**Authors:** İlker UYAR, Zeynep ALTUNTAŞ, Sıddıka FINDIK, Mehmet Emin Cem YILDIRIM, Serhat YARAR, Murat AKTAN, Ahmet AVCI

**Affiliations:** 1 Department of Plastic, Reconstructive and Aesthetic Surgery, Faculty of Medicine, İzmir Katip Çelebi University, İzmir, Turkey; 2 Department of Plastic, Reconstructive and Aesthetic Surgery, Faculty of Medicine, Necmettin Erbakan University, Konya, Turkey; 3 Department of Pathology, Faculty of Medicine, Necmettin Erbakan University, Konya, Turkey; 4 Department of Plastic, Reconstructive and Aesthetic Surgery, Faculty of Medicine, İstinye University, İstanbul, Turkey; 5 Plastic, Reconstructive and Aesthetic Surgery Clinic, Konya City Hospital, Konya, Turkey; 6 Department of Histology and Embryology, Faculty of Medicine, Necmettin Erbakan University, Konya, Turkey; 7 Department of Biomedical Engineering, Faculty of Medicine, Necmettin Erbakan University, Konya, Turkey

**Keywords:** Experimental study, mesenchymal stem cell, platelet-rich plasma, tendon healing

## Abstract

**Background/Aim:**

The objective of this study was to investigate the effects that the application of mesenchymal stem cells (MSCs) and platelet-rich plasma (PRP) following tendon repair have on the strength and healing of the tendon and also to examine the possible mechanisms of action that take place.

**Materials and methods:**

The Achilles tendons of 80 rats were repaired and divided into eight groups. Following the repairs, MSCs obtained from humans were injected into the rat tendons in groups 1 and 2, a combination of MSCs from humans and PRP from rats was injected into the tendons in groups 3 and 4, and PRP from rats was injected into the tendons in groups 5 and 6. These procedures all took place simultaneously. Groups 7 and 8 did not receive any injections following the repairs. The rats were sacrificed at the end of the first and second months following the procedures, and biomechanical and histopathological analyses were performed.

**Results:**

Inflammatory cell density increased most significantly in the combined group when compared to the first and second months. The fibroblast density on the tendon repair region was significantly lower in the second-months groups of each intervention compared to their first-month groups (p = 0.001). For the analysis of the maximum tensile breaking force, the behaviors of the groups over time were significant when compared to the control groups (p = 0.0015). Also, the mean maximum breaking force in the combined group was statistically significantly higher at the end of the second month than at the end of the first month (p = 0.0008).

**Conclusion:**

The combination therapy increased tendon strength force. This combination therapy can make a positive contribution to the healing of tendons after surgery.

## 1. Introduction

Recently, biological treatments have been explored with regard to tendon healing, to speed up healing and increase the tension of the tendon. On the basis of the hypothesis proposed in our study, if tendon healing can be accelerated, adhesions can be prevented by starting tension movements earlier [1].

Treatment that is based on mesenchymal stem cells (MSCs) positively affects tissue healing through three different mechanisms [2]. First, MSCs modulate the immune response and provide these immunomodulatory effects through direct cell-to-cell interaction or soluble immunosuppressive factors [3]. Second, MSCs release various cytokines, growth factors, and chemokines. These exert their effects by inhibiting apoptosis and promoting adjacent cell proliferation [4]. Third, MSCs may have the potential to differentiate into various cells, some of which include bone, cartilage, tendon, adipose, bone marrow stroma, and muscle cells [5].

Platelet-rich plasma (PRP) is a plasma component obtained with full blood centrifugation, which includes a higher concentration of platelets than full blood [6,7]. This plasma has numerous growth factors, enabling the use of PRP injections in the treatment of various musculoskeletal diseases. It has been reported that growth factors that are considered to affect the healing process might, because of their effects in increasing tendon regeneration, be able to be used in the treatment by giving direct local injections to the lesion sites [8]. There are studies in the literature that show the positive effects that PRP and mesenchymal stem cell injections have on tendon healing [9]. However, there have been no controlled experimental studies that have demonstrated and compared these effects [10].

The aim of this study was to reveal, separately, the effects of PRP, MSCs, and therapies that combine MSCs and PRP on the healing of the Achilles tendons of the rats used in the experiment and to compare them through evaluations during the follow-up period.

## 2. Materials and methods

The approval of Necmettin Erbakan University KONÜDAM Experimental Medicine Application and Research Center was obtained for this study (decision number: 2017-D20). This experimental animal investigation was carried out according to a high ethical standard.

**Figure 1:**
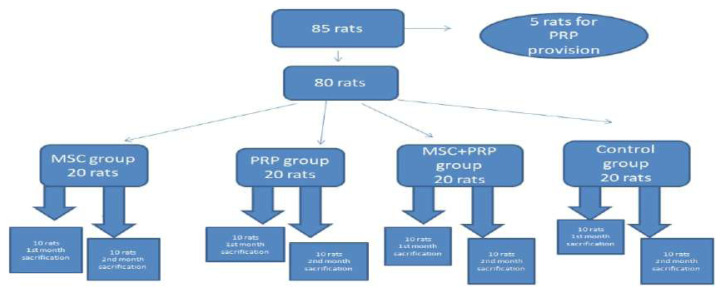
The view of the methods for all groups. After the repair procedure, 0.1 cc MSCs were injected in Groups 1 and 2; a combination of 0.1 cc MSCs and 0.1 cc PRP were injected in Groups 3 and 4; 0.1 cc PRP were injected in Groups 5 and 6; simultaneously. Group 7 and 8 did not receive any injection following surgical stress as control groups.

In our study, rats of the Wistar Albino breed, the average weight of 350–500 g, the mean age of 8–12 months, and the male gender were used. A total of 85 rats were used in the study with five being used only as donors, and the rats were randomly divided into eight groups using a closed envelope method with 10 rats in each group. Before surgery, 30 mg/kg cefazolin sodium (İespor®, İ.E. Ulagay, Turkey) was administered intraperitoneally to the rats. Then 35 mg of ketamine HCL (Ketalar®, Pfizer, USA) and 5 mg of xylazine (Rompun®, Bayer, Germany) per kg were administered intraperitoneally and general anesthesia was provided. The left tendons of the rats in all the groups were cut during the same session and repaired using the modified Kessler method [1]. After the repairs, 0.1 cc of MSCs obtained from humans was injected in groups 1 and 2 (the recovery of the stem cells was performed according to the use of human-derived MSCs in the rat tendon injury model described by Lee et al., stem cell extraction is further detailed below), a combination of 0.1 cc of MSCs from humans plus 0.1 cc of PRP obtained from the rats was injected in groups 3 and 4, and 0.1 cc of PRP from rats was injected in groups 5 and 6. These injections were performed simultaneously into the repaired Achilles tendons of the rats. The rats in two other groups, 7 and 8, did not receive any injections following surgery, and these groups were used as controls (Figure 1).

The rats in groups 1, 3, 5, and 7 were sacrificed at the end of the first month, and the rats in groups 2, 4, 6, and 8 at the end of the second month. A piece of bone was removed from the calcaneus and a piece of muscle from the triceps surae in all groups during tendon excision. Biomechanical and histopathological analyses were performed on the left Achilles tendons.

Based on the principle that there is no biomechanical difference between fresh frozen tendons and never waited tendons, the tendons obtained from groups 1, 3, 5, and 7 were stored in the laboratory setting at −23 °C until the biomechanical analysis was performed [11].

### 2.1. Stem cell preparation

**Figure 2:**
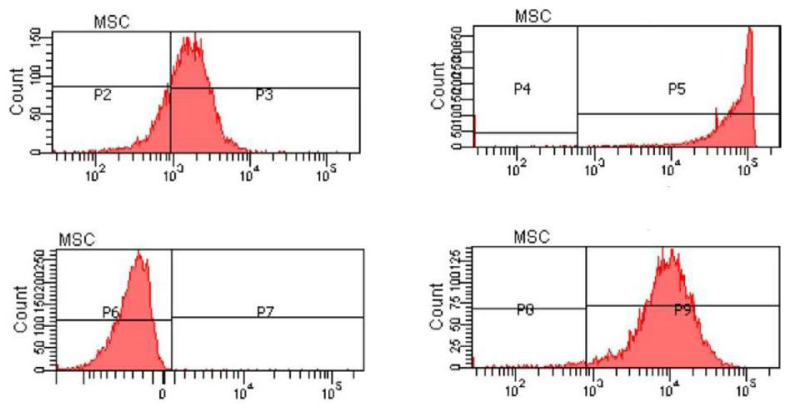
Flowcytometry image of the behavior of CD marker marked mesenchymal stem cells in P blocks, immunophenotyping of mesenchymal stem cells by flowcytometry. Apoptotic effects on the cells. Upper left is for CD105, upper right is for CD44, lower left is for CD45, lower right is for CD90.

Rats are not preferred as a source of stem cells, since rats do not have enough stem cell sources and the aim of the study was to obtain many stem cells. The recovery of the stem cells was performed according to the use of human-derived MSCs in the rat tendon injury model described by Lee et al. [12]. The lipoaspirate for the isolation of the MSCs was obtained from the subcutaneous tissues of people who had been informed about the subject, had given consent, and had no health problems; the procedures were performed in an operating room setting. The lipoaspiratee was irrigated witphosphate-buffereded saline, 1% bovine serum albumin, and 0.025% collagenase type 1, and the isolated stromal vascular fraction were used in the study. The MSCs were evaluated in terms of appearance, viability, and potent. This evaluation involved examining cellular morphology, doubling times, karyotypes, cell surface markers (CD44, CD90, CD105, and CD45), and biological functions such as the growth factor releases and immunosuppressive activities. The cells that showed less than 80% viability and less than 1% CD45 positivity were excluded from the study (Figure 2).

### 2.2. PRP preparation

Before surgery, 30 mg/kg cefazolin sodium (İespor®, İ.E. Ulagay, Turkey) was administered intraperitoneally to the rats. Then 35 mg of ketamine HCL (Ketalar®, Pfizer, USA) and 5 mg of xylazine (Rompun®, Bayer, Germany) per kg was administered intraperitoneally and general anesthesia was provided. For harvesting the PRP, 5 cc of intracardiac blood was drawn from five donor rats under general anesthesia. The PRP was obtained using Smith and Nephew Prosys (PRP) biokits (Smith and Nephew, England) and table type VS-5000i2 centrifugation devices (Nüve, Turkey) at 3000 rpm for three 6 min. Platelet measurements were made in the PRP using a biochemical analysis device (Siemens Adviva 212i, Germany).

### 2.3. Histopathological evaluation

The examination was carried out by two different pathologists. The excised tissues were fixed in 10% formaldehyde for evaluation under a light microscope. Sections of 4–5 μm were taken from the samples and embedded in paraffin blocks for hematoxylin and eosin (H&E), masson trichrome (MTC), and Alcian blue staining. In light microscopy, the inflammatory cell density, type of inflammatory cells, and vascularization were evaluated in the H&E stained slides, and the fibroblast density was assessed in the MTC stained slides.

Inflammation and fibrosis scoring methods: 0 = none, 1 = low, 2 = moderate, 3 = high.

Vascularization (at 1 large magnification): 0–5 capillaries = low – 1, 6–10 capillaries = moderate – 2, more than 10 capillaries = high – 3.

### 2.4. Biomechanical tests

Biomechanical tests were performed in the Biomechanics Laboratory. Tendon tensile movements (modulus of elasticity, stiffness, energy absorption capabilities, maximum loading amount, breaking loads) were examined. The maximum tensile strength of the tendons was recorded in Newtons. Before the tests were done, the tendons were waited to solve at room temperature and moistened with ringer lactate solution intermittently to prevent drying [11]. The tests were performed with a tensile device (Autograph AG-IS 100 kN Shimadzu Co. Kyoto, Japan) by giving a tensile load at a rate of 1 mm/min. The tensile movements of the tendons were examined. All breakages at the end of the tests occurred in the regions that had been cut and then repaired and in Achilles tendons that had been inserted into the device from the musculotendinous junction in the proximal region and the calcaneus in the distal region.

### 2.5. Statistical analysis

**Figure 3:**
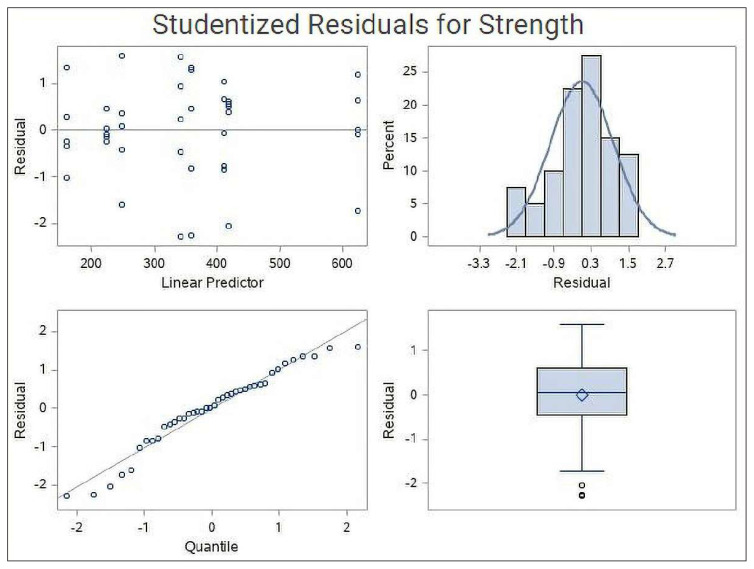
Hypothetical reviews of tendon analyzes.

Variables were analyzed with ANOVA-type statistic. Numerical variables are given as a mean (standard deviation), and categorical variables are presented as numbers (percentages). Ordinal categorical variables were analyzed using the R 3.4.3 nparLD package and dichotomous variables were analyzed using a generalized linear model. The analyses were performed with R 3.4.3 software for the ordinal variables and SAS University Edition 9.4 software (SAS, USA) for the ordinal variables [13]. The results were evaluated at a 95% confidence interval and p < 0.05 level of significance (Figure 3).

## 3. Results

### 3.1. Histopathological findings

**Figure 4:**
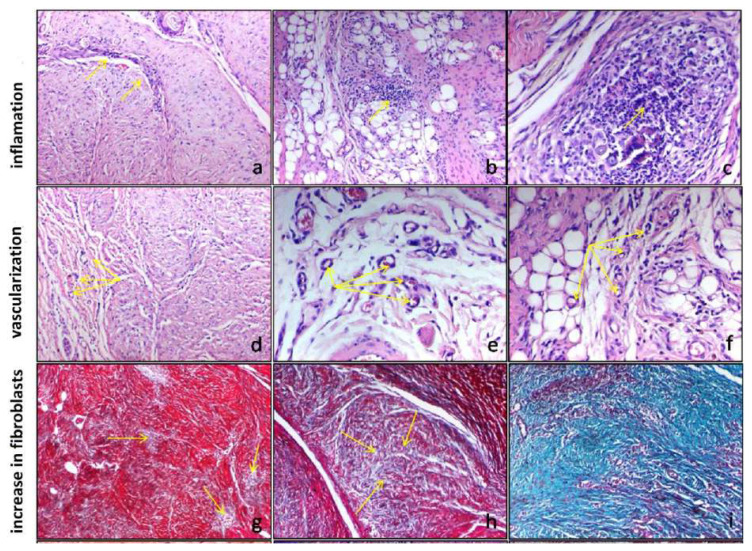
Histopathologic images. a–b–c: Inflammation low-moderate-high (x100,100,200 H&E) d–e–f: Vascularization low-moderate-high (x40,200,200 H&E) g–h–i: Fibroblast increase low-moderate-high (x100 Masson’s Trichrome)

Inflammatory cell density were analyzed as low–moderate–high (Figure 4a–4c), vascularization was analyzed as low–moderate–high (Figure 4d–4f), and fibroblast density was analyzed as low–moderate–high (Figure 4g–4i) in the histopathological findings (Figure 4).

#### 3.1.1. Inflammatory cell density

At the end of the first month, the inflammatory cell density was low in 40%, moderate in 40%, and high in 20% of the rats in group 1 (MSCs), low in 20%, moderate in 40%, and high in 40% of the rats in group 3 (combined). The inflammatory cell density was moderate in 60% and high in 40% of the rats in group 5 (PRP). However, the inflammatory cell density was high in all of the rats in the control group.

At the end of the second month, the inflammatory cell density was low in 40%, moderate in 20%, and high in 40% of the tissues in group 2 (MSCs) and was high in all tissues in group 4 (combined). The inflammatory cell density was low in 20%, moderate in 40%, and high in 40% of the tissues in group 6 (PRP) and low in 20%, moderate in 60%, and high in 20% of the tissues in group 8 (control) (Table 1).

**Figure 5:**
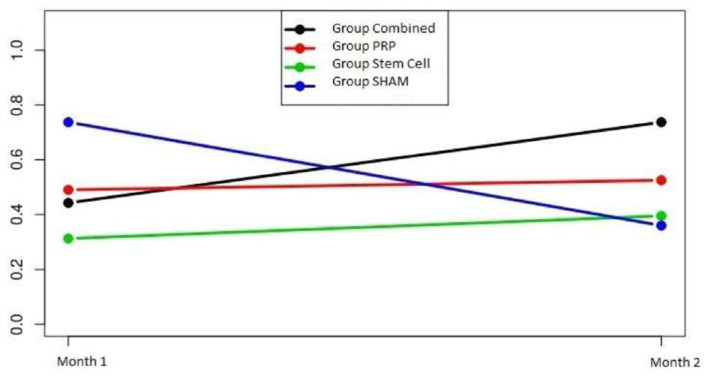
Inflammatory cell density group-time plot of the groups. The inflammatory cell density was highest in the control group at the end of the first month, but was lowest in that group at the end of the second month, while being higher in all other groups. Inflammatory cell density increased most significantly in the combined group when compared at the first and second months.

In the analysis of the inflammatory cell density, the group-time interaction was significant in all groups (p = 0.038). However, the changes in inflammatory cell densities over time were different among the groups (Figure 5).

#### 3.1.2. Vascularization

At the end of the first month, the vascularization was low in 20%, moderate in 60%, and high in 20% of the tissues in group 1 (MSCs), while it was moderate in 20% and high in 80% of the tissues in group 3 (combined). The vascularization was moderate in 40% and high in 60% of the tissues in group 5 (PRP) and was low in 20%, moderate in 40%, and high in 40% of the tissues in group 7 (control).

At the end of the second month, the vascularization was moderate in 60% and high in 40% of the tissues in group 2 (MSCs), while it was moderate in 20% and high in 80% of the tissues in group 4 (combined). The vascularization was low in 20%, moderate in 40%, and high in 40% of the tissues in group 6 (PRP) and was low in 20%, moderate in 60%, and high in 20% of the tissues in group 8 (control) (Table 2).

**Figure 6:**
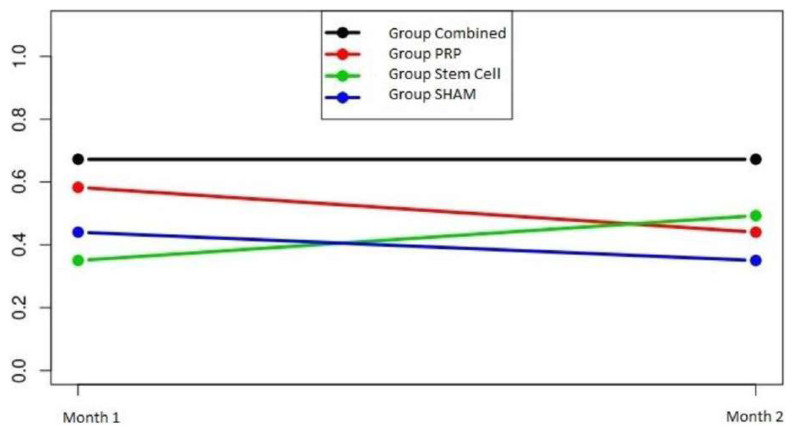
Vascularization group-time plot of the groups. The vascularization decreased in the control and PRP groups but increased at the end of the second month in the group that received MSCs obtained from humans. In the combined group, the vascularization level was the highest after the first month and did not change at the end of the second month.

In the analysis of the vascularization, the group-time interaction was not significant in any of the groups (p = 0.49). The group and time effects were also not significant (p = 0.13 and p = 0.76, respectively), and the levels of vascularization remained the same over time in all the groups (Figure 6).

#### 3.1.3. Fibroblast density

At the end of the first month, the fibroblast density was low in 20%, moderate in 20%, and high in 60% of the tissues in group 1 (MSCs), while it was low in 20%, moderate in 40%, and high in 40% of the tissues in group 3 (combined). For the tissues in group 5 (PRP), the fibroblast density was moderate in 80% and high in 20%, and the fibroblast and collagen densities were moderate in 20% and high in 80% of the tissues in group 7 (control).

At the end of the second month, the fibroblast density was low in 20%, moderate in 60%, and high in 20% of the tissues in group 2 (MSCs), while it was moderate in 80% and high in 20% of the tissues in group 4 (combined). For the tissues in group 6 (PRP), the fibroblast density was low in 20% and moderate in 80%, and it was low in 60% and moderate in 40% of the tissues in group 8 (control) (Table 3).

**Figure 7:**
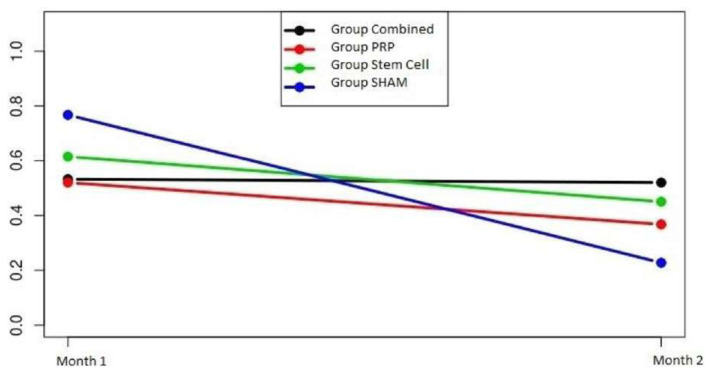
Fibroblast density-time plot of the groups. Fibroblast density levels decreased from the first month and toward the second month in all groups. This decrease was slower in the combined group than in the other groups.

In the fibrosis analysis, the group*time interaction was significant (p = 0.06). The changes in cell densities of the groups over time were not different from each other. The time effect was significant (p = 0.001). Fibrosis values of the groups were not different from each other (p = 0.79) (Figure 7).

### 3.2. Biomechanical findings

**Figure 8:**
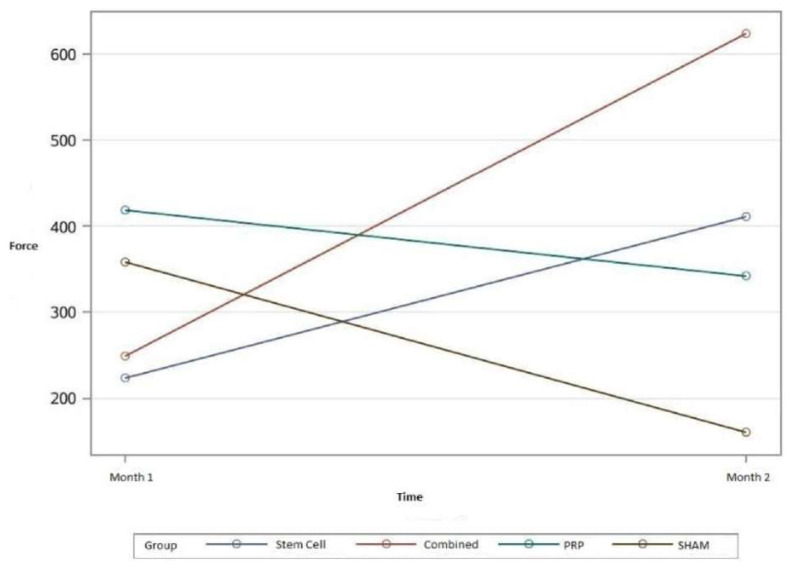
Maximum breaking force plot of the groups. The mean maximum breaking force in the combined group was statistically significantly higher at the end of the second month than at the end of the first month. The mean maximum breaking force in the MSC group was higher at the end of the second month than at the end of the first month. The mean tendon forces that were measured in the control and PRP groups at the end of the first month were higher than the mean forces measured at the end of the second month.

In the maximum breaking force analysis, the behaviors of all groups over time were statistically significant when compared to the control group (p = 0.0015) (Figure 8).

The mean maximum breaking force of the tendons was found to be 223.51 in group 1, 410.90 in group 2, 248.96 in group 3, 623.69 in group 4, 418.40 in group 5, 341.86 in group 6, 358.26 in group 7, and 160.55 in group 8. The standard deviation was calculated as 71.8196 in all groups (Table 4).

A comparison of the changes between the groups in terms of the mean maximum breaking force of the tendons, according to months, found that this figure was lower in group 1 than in group 2, but the difference was not statistically significant (p = 0.07). However, the mean maximum breaking force was lower in group 3 than in group 4, and this difference was statistically significant (p = 0.0008). The mean maximum breaking force was higher in group 5 than in group 6 (p=0.46), and the mean maximum breaking force was higher in group 7 than in group 8, but neither of these differences was statistically significant (p = 0.06).

The mean maximum breaking force of the tendons, according to months, in all groups was also compared to the control group. At the end of the first month, the mean maximum breaking force was lower in group 1 than in group 7, but the difference was not statistically significant (p = 0.19). The mean maximum breaking force was also lower in group 3 than in group 7, and the difference was not statistically significant (p = 0.28). While the mean maximum breaking force was higher in group 5 than in group 7, this difference was not statistically significant (p = 0.55) either.

At the end of the second month, the mean maximum breaking force was higher in group 2 than in group 8, and this difference was statistically significant (p = 0.01). In addition, at the end of the second month, the mean maximum breaking force was higher in group 4 than in group 8, and again the difference was statistically significant (p < 0.0001). However, at the end of the second month, although the mean maximum breaking force was higher in group 6 than in group 8, the difference was not statistically significant (p = 0.08).

## 4. Discussion

After tendon repairs have been completed, extrinsic and intrinsic healing is observed and a number of different treatment methods have recently been investigated to achieve a balance between the two types of healing [14–18].

According to the literature, inflammatory cells and high amounts of fibrosis have been found around the suture material in the early period after tendons are repaired [19,20].

Cell migration occurs as part of the inflammatory process of tendon healing. Growth factors and cytokines also stimulate fibroblasts, and the cell/matrix ratio is regulated by the new collagen fibers that are formed. During the later period after tendon repairs, the numbers of cells decrease, and apoptosis is one possible reason for this decrease [19,20]. In our study, the inflammatory cell density was highest in the control group at the end of the first month, but was lowest in that group at the end of the second month, while being higher in all other groups. Inflammatory cell density increased most significantly in the combined group when compared to the first and second months. With regard to the analysis of the inflammatory cell density, the group-time interaction was significant (p = 0.038), and the changes in the inflammatory cell densities over time were different among the groups. The reason for the rapid decrease in the inflammatory cells in the control group is that the apoptosis process occurs more quickly in this group. The increase that occurred in the inflammatory cells in the combined group toward the end of the second month when compared to that of the other groups may cause the density of fibroblasts to be higher in this region. This positive contribution may, then, allow the maximum breaking force to reach a higher level in the combined group than in the others.

In the analysis of the vascularization, the group-time interaction and the group and time effects were not statistically significant. However, the vascularization decreased in the control and PRP groups but increased at the end of the second month in the group that received MSCs obtained from humans. In the combined group, the vascularization level was the highest after the first month and did not change at the end of the second month. One of the reasons for the increase in the mean maximum breaking force in the group that received MSCs obtained from humans and in the combined group might be this vascularization pattern.

In our study, fibroblast density levels decreased from the first month and toward the second month in all groups. This decrease was slower in the combined group than in the other groups, and the decrease was very significant in the control group. The reason for this pattern may be that the apoptosis process extended over time in the combined group. In our study, time effect was significant in the fibroblast density analysis (p = 0.001). We believe that the pattern of delayed apoptosis in the combined group keeps the fibroblast density high for a long time, and this positively contributes to the tension force of the tendon.

Although studies have not found a statistically significant difference between the control and MSC groups in terms of biomechanical evaluation, significant results have been obtained in histologic evaluations [21–23]. In our study, in terms of biomechanical evaluations, the behaviors of the groups over time were different in the analysis of the maximum breaking force of the tendons, and the difference was statistically significant (p = 0.0015). The mean maximum breaking force in the combined group was statistically significantly higher at the end of the second month than at the end of the first month (p = 0.0008). In addition, the mean maximum breaking force in the MSC group was higher at the end of the second month than at the end of the first month, but this difference was not statistically significant. The mean tendon forces that were measured in the control and PRP groups at the end of the first month were higher than the mean forces measured at the end of the second month. In our daily observations, we found that the rats in all groups were more likely to protect their left legs, on which the surgeries had been performed, and to hold them in the resting position in the postoperative period. The reason for the decreased maximum breaking forces in the tendons at the end of the second month in the control and PRP groups might be that, during the healing process, the rats did not put load on the legs on which the surgeries had been performed. However, no negative effects were found from this in the MSC and combined groups, and higher values were measured in these groups at the end of the second month, with this difference being more prominent in the combined group. We believe that this is due to the synergistic effect of the combination of MSCs and PRP and maybe because MSCs tend to have strong effects with regard to growth factors. In spite of the tendency of the rats to protect the legs on which the surgeries had been performed, the MSCs and the combination of MSCs and PRP positively contributed to healing. Also, this contribution was more prominent in the combined group.

Uysal et al. conducted a study on the Achilles tendons of rabbits in 2012, using autologous PRP and PRP+stem cell combinations, and obtained positive results in terms of tensile strength in the stem cell group [24]. However, a disadvantage to this study is that it has no control group or stem cell group. An important difference between this study and ours is that the stem cell is heterologous in ours and includes all groups.

Yuksel et al. used autologous bone marrow stem cells and autologous PRP on the Achilles tendons of rats in a study done in 2016, and successful results were obtained [25]. However, this study has no group in which stem cells and PRP treatment are combined.

In a study conducted in 2013 by Martinello et al., autologous treatments using PRP and MSCs were applied to the flexor tendons of sheep, and successful results were obtained [26]. However, although this study includes stem cells, PRP, and combined groups, there is no control group. Our study differs from other studies because it contains all of the following groups and combinations: a control group, an MSC group, a PRP group, and combined groups (MSCs + PRP). In addition, our study is similar to other studies in terms of the use of autologous PRP but differs from other studies in terms of the production of stem cells.

Also, MSCs in our study were not obtained from the rats but from a different species, and no study reporting on combined treatment using MSCs from a different species is available in the literature. In addition, having a donor area and providing more MSCs are important advantages that result from this method. However, the antigenic structure of the stem cell negatively affects the survival time in the application area, and this effect can be considered to be a disadvantage of the study. Another limitation of the study is that the future levels of the mesenchymal stem cell densities in the tissue are unknown, and our study includes no review for this.

With regard to the study performed by Lee et al., the xenogenic stem cells that were obtained can maintain their viability for about four weeks [12]. With the desensitization studies that will be carried out in the future, we think that the viability period of xenogenic stem cells might be extended, and that these stem cells may be useful in future experimental trials.

## 5. Conclusion

In conclusion, our study found that at the end of the second month after the rats’ Achilles tendons had been repaired, there was a higher mean maximum breaking force in the group in which the combination of the MSCs and PRP was used than in any of the other groups. In addition, this difference was found to be statistically and histologically significant. Based on this result, we believe that combination therapy using MSCs and PRP will guide further clinical studies and influence the tendon repair process in the future.

## Figures and Tables

**Table 1:** Inflammatory cell density in groups.

Groups	Inflammatory cell density								
	Month 1			Month 2			Group	Month	Group-time interaction
	Low	Moderate	High	Low	Moderate	High			
MSCs	4 (%40)	4 (%40)	2 (%20)	4 (%40)	2 (%20)	4 (%40)	ANOVA-type statistic: 2.17 Df: 2.79 P: 0.09	ANOVA-type statistic: 0.01 Df: 1 P: 0.91	ANOVA-type statistic: 3.03 Df: 2.48 P: 0.038
Combined	2 (%20)	4 (%40)	4 (%40)	0 (%0)	0 (%0)	10 (%100)			
PRP	0 (%0)	6 (%60)	4 (%40)	2 (%20)	4 (%40)	4 (%40)			
Control	0 (%0)	0 (%0)	10 (%100)	2 (%20)	6 (%60)	2 (%20)			

**Table 2:** Vascularization in groups.

Groups	Vascularization								
	Month 1			Month 2			Group	Month	Group-time interaction
	Low	Moderate	High	Low	Moderate	High			
MSCs	2 (%20)	6 (%60)	2 (%20)	0 (%0)	6 (%60)	4 (%40)	ANOVA-type statistic: 1.96 Df: 2.64 P: 0.12	ANOVA-type statistic: 0.09 Df: 1 P: 0.75	ANOVA-type statistic: 0.74 Df: 2.23 P: 0.49
Combined	0 (%0)	2 (%20)	8 (%80)	0 (%0)	2 (%20)	8 (%80)			
PRP	0 (%0)	4 (%40)	6 (%60)	2 (%20)	4 (%40)	4 (%40)			
Control	2 (%20)	4 (%40)	4 (%40)	2 (%20)	6 (%60)	2 (%20)			

**Table 3:** Fibroblast density in groups.

Groups	Fibroblast density								
	Month 1			Month 2			Group	Month	Group- time interaction
	Low	Moderate	High	Low	Moderate	High	ANOVA-type statistic: 0.24 Df: 2.06 P: 0.79	ANOVA-type statistic: 10.3 Df: 1 P: 0.001	ANOVA-type statistic: 2.78 Df: 1.99 P: 0.06
MSCs	2 (%20)	2 (%20)	6 (%60)	2 (%20)	6 (%60)	2 (%20)
Combined	2 (%20)	4 (%40)	4 (%40)	0 (%0)	8 (%80)	2 (%20)
PRP	0 (%0)	8 (%80)	2 (%20)	2 (%20)	8 (%80)	0 (%0)
Control	0 (%0)	2 (%20)	8 (%80)	6 (%60)	4 (%40)	0 (%0)

**Table 4:** The mean maximum breaking force and standard deviation of the groups.

Groups	The mean maximum breaking force		Df	t value	P
	Month 1	Month 2			
**MSCs**	223.51 ± 39.22	410.90 ± 120.98	32	−1.84	0.07
**Combined**	248.96 ± 167.06	623.69 ± 157.42	32	−3.69	0.0008
**PRP**	418.40 ± 164.58	341.86 ± 213.57	32	0.75	0.46
**Control**	358.26 ± 220.50	160.55 ± 127.05	32	1.95	0.06

## References

[1] Andarawis-Puri N Flatow EL Soslowsky LJ Tendon basic science: development, repair, regeneration, and healing Journal of Orthopaedic Research 2015 33 6 780 784 10.1002/jor.22869 25764524 PMC4427041

[2] Wang J Liao L Tan J Mesenchymal-stem-cell-based experimental and clinical trials: current status and open questions Expert Opinion on Biological Therapy 2011 11 7 893 909 10.1517/14712598.2011.574119 21449634

[3] Uccelli A Moretta L Pistoia V Mesenchymal stem cells in health and disease Nature Reviews Immunology 2008 8 9 726 736 10.1038/nri2395 19172693

[4] Kinnaird T Stabile E Burnett MS Lee CW Barr S Marrow-derived stromal cells express genes encoding a broad spectrum of arteriogenic cytokines and promote in vitro and in vivo arteriogenesis through paracrine mechanisms Circulation Research 2004 94 5 678 685 10.1161/01.RES.0000118601.37875.AC 14739163

[5] Pittenger MF Mackay AM Beck SC Jaiswal RK Douglas R Multilineage potential of adult human mesenchymal stem cells Science 1999 284 5411 143 147 10.1126/science.284.5411.143 10102814

[6] Paoloni J De Vos RJ Hamilton B Murrell GA Orchard J Platelet-rich plasma treatment for ligament and tendon injuries Clinical Journal of Sport Medicine 2011 21 37 45 10.1097/JSM.0b013e31820758c7 21200169

[7] Ince B Yildirim MEC Dadaci M Avunduk MC Savaci N Comparison of the efficacy of homologous and autologous platelet-rich plasma (PRP) for treating androgenic alopecia Aesthetic Plastic Surgery 2018 42 1 297 303 10.1007/s00266-017-1004-y 29101437

[8] Rees JD Maffulli N Cook J Management of tendinopathy The *American Journal* of *Sports Medicine* 2009 37 1855 67 10.1177/0363546508324283 19188560

[9] Yildirim MEC Ince B Uyanik O Okur MI Dadaci M Development of hyperalgesia in patients treated with autologous platelet rich plasma due to androgenetic alopecia Selçuk Tıp Dergisi 2018 34 3 90 93 10.30733/std.2018.01051

[10] Hoffmann A Gross G Tendon and ligament engineering in the adult organism: mesenchymal stem cells and gene therapeutic approaches *International Orthopaedics* 2007 31 791 797 10.1007/s00264-007-0395-9 17634943 PMC2266662

[11] Bhatia D Tanner KE Bonfield W Citron ND Factors affecting the strength of flexor tendon repair The Journal of *Hand Surgery*: British & European Volume 1992 17 5 550 552 10.1016/s0266-7681(05)80240-2 1479249

[12] Lee SY Kwon B Lee K Son YH Chung SG Therapeutic mechanisms of human adipose-derived mesenchymal stem cells in a rat tendon injury model The *American Journal* of *Sports Medicine* 2017 45 6 1429 1439 10.1177/0363546517689874 28291954

[13] Brunner E Domhof S Langer F Nonparametric Analysis of Longitudinal Data in Factorial Experiments NY, USA Wiley 2002

[14] Karacor Z Moran SL The effect of 5 FU on the expression of transforming growth factor beta-1 (tgf-1) in cultured tendon cells Acta Medica Anatolia 2014 4 138 142 10.15824/actamedica.75043

[15] Zhao C Zobitz ME Sun YL Predmore KS Amadio PC Surface treatment with 5-fluorouracil after flexor tendon repair in a canine in vivo model The Journal of *Bone* and *Joint Surgery* 2009 91 11 2673 2682 10.2106/JBJS.H.01695 19884442 PMC2767124

[16] Zhao C Chieh HF Bakri K Ikeda J Sun YL The effects of bone marrow stromal cell transplants on tendon healing in vitro Medical *Engineering* and *Physics* 2009 31 10 1271 1275 10.1016/j.medengphy.2009.08.004 19736035 PMC3898667

[17] Maruyama M Wei L Thio T Storaci HW Ueda Y The effect of mesenchymal stem cell sheets on early healing of the achilles tendon in rats *Tissue Engineering*, *Part A* 2020 26 3–4 206 213 10.1089/ten.TEA.2019.0163 31608794

[18] Türkmen F Özer M Kaçıra BK Korucu İH Göncü G Bilateral Spontaneous Rupture of Achilles Tendons İn Absence of Risk Factors Selcuk Medical Journal 2019 35 3 203 206 10.30733/std.2019.00856 (in Turkish with an abstract in English)

[19] Lui PP Cheuk YC Hung LK Fu SC Chan KM Increased apoptosis at the late stage of tendon healing *Wound Repair* and *Regeneration* 2007 15 702 707 10.1111/j.1524-475X.2007.00276.x 17971016

[20] Hapa O Cakici H Gideroğlu K Ozturan K Kukner A The effect of ethanol intake on tendon healing: a histological and biomechanical study in a rat model Archives of *Orthopaedic* and *Trauma Surgery* 2009 129 1721 1726 10.1007/s00402-009-0877-x 19381661

[21] Awad HA Boivin GP Dressler MR Smith FN Young RG Repair of patellar tendon injuries using a cell-collagen composite The *Journal of Orthopaedic Research* 2003 21 420 431 10.1016/S0736-0266(02)00163-8 12706014

[22] Chong AK Ang AD Goh JC Hui JH Lim AY Bone marrow-derived mesenchymal stem cells influence early tendon-healing in a rabbit achilles tendon model The Journal of *Bone* and *Joint Surgery* 2007 89 74 81 10.2106/JBJS.E.01396 17200313

[23] Young RG Butler DL Weber W Caplan AI Gordon SL Use of mesenchymal stem cells in a collagen matrix for achilles tendon repair The *Journal of Orthopaedic Research* 1998 16 406 413 10.1002/jor.1100160403 9747780

[24] Uysal C Tobita M Hyakusoku H Mizuno H Adipose-derived stem cells enhance primary tendon repair: biomechanical and immunohistochemical evaluation Journal of Plastic, Reconstructive and Aesthetic Surgery 2012 65 12 1712 1719 10.1016/j.bjps.2012.06.011 22771087

[25] Yuksel S Guleç MA Gultekin MZ Adanır O Caglar A Comparison of the early period effects of bone marrow-derived mesenchymal stem cells and platelet-rich plasma on the achilles tendon ruptures in rats *Connective Tissue Research* 2016 57 5 360 373 10.1080/03008207.2016.1189909 27191749

[26] Martinello T Bronzini I Perazzi A Testoni S De Benedictis GM Effects of in vivo applications of peripheral blood-derived mesenchymal stromal cells (PB-MSCs) and platelet-rich plasma (PRP) on experimentally injured deep digital flexor tendons of sheep The *Journal of Orthopaedic Research* 2013 31 2 306 314 10.1002/jor.22205 22893604

